# Supplementary material for: Computing the Parameter Values for the Emergence of Homochirality in Complex Networks
Source: Life (Basel). 2019 Sep 15;9(3):74. doi: 10.3390/life9030074 (PMC6789494; doi:10.3390/life9030074)
Supplement: Supplementary file 1 [file life-09-00074-s001.pdf]

Supplementary Materials: Computing the parameter values for the emergence of homochirality in complex networks

Andrés Montoya [1,‡], Elkin Cruz [2,‡] and Jesús Ágreda [2,‡*]

[1] Departamento de Matemáticas, Universidad Nacional de Colombia; jamontoyaa@unal.edu.co
[2] Departamento de Química, Universidad Nacional de Colombia; elacruzca@unal.edu.co ; jagreda@unal.edu.co
* Correspondence: jagreda@unal.edu.co; Tel.: +57-1-3165000 Ext. 14431
‡ These authors contributed equally to this work.

This document presents information that is either complementary to the main paper, or too long to be in it. The document is divided into the following sections: where to find the code used in the calculations, typical examples of the Listanalchem outputs for the models studied in the paper, and the Iwamoto model under imperfect conditions figure, which is not in the main document.

1. The software developed and used in the paper

The algorithm to study the stability of the models proposed to explain the origin of biological homochirality, and developed in the paper, was implemented in the computer software Listanalchem, option six. The software is available at:

`http://ciencias.bogota.unal.edu.co/gruposdeinvestigacion/grupo-de-estudios-para-la-remediacion-y-mitigacion-de-impactos-negativos-al-ambiente-germina/software/`

or

`https://gitlab.com/homochirality`

The simulations of the models (the numerical solution of the respective ordinary differential equations) were made using the software Chemkinlator, also available at the same URL given above.

2. Inputs and outputs of Listanalchem for the models studied in the paper

In the following subsections, a typical output example of Listanalchem, option six, is presented for the four models studied in the paper. The use of random sampling, in some steps of the algorithm, makes the numerical part of the outputs calculated by Listanalchem different every single time the program is executed. Thus, if you run Listanalchem on the models studied in the paper, you may get different quantitative results from those shown in figures 1, 2, and 3 of the paper. However, only the quantitative aspects will be different, but not the qualitative ones.

Models in Listanalchem are defined as plain text files. A model must define a name, a set of species, and a set of reactions. The models studied in the paper are in the folder "models" of the program Listanachem after it is installed. Please, see the README.md file, to get instructions to install and run the program. The analysis of a network can be made writing the command line:

```
python -m listanalchem --model models/Model-name.py
```

In the next subsections, and for every model analyzed in the paper, the input and the output files are presented.

2.1. The Calvin model

2.1.1. The input file

```
 # run with: "python -m listanalchem --model models/Calvin-pseudoquiral.py"
modelname = ’Calvin - pseudoquiral’
species = [’L1’, ’D1’, ’L2’, ’D2’]
reactions = [
    ’L1 + L2 -> 2L2’,  # 0
    ’D1 + D2 -> 2D2’,  # 1
    ’2L2 -> L1 + L2’,  # 2
    ’2D2 -> D1 + D2’,  # 3
    ’L1 -> D1’,  # 4
    ’D1 -> L1’,  # 5
    ’L1 -> L2’,  # 6
    ’D1 -> D2’,  # 7
    ’L2 -> L1’,  # 8
    ’D2 -> D1’,  # 9
]

dual_pairs = [(0, 1), (2, 3), (6, 7), (8, 9)]

analyses = {
    "sna": {
        "enabled": True,
        "dual-pairs-in-ec": True,
        # "instability-heuristic": "trace-determinant",
        # "instability-heuristic": "characteristic-polynomial",
        "instability-heuristic": "mineurs",
        "sum-mineurs": True,
        "max-mineur-search-stop": 5,
        "simplification-tries": 1000,
        "num-samples": 10,
        "samples-folder": ’samples/calvin-pseudoquiral’,
    },
    "frank-pseudoquiral": {
        "enabled": True,
        "enantiomeric-pairs": [(0, 1), (2, 3)],
        "dual-pairs-in-ec": True,
        # "instability-heuristic": "trace-determinant",
        # "instability-heuristic": "characteristic-polynomial",
        "instability-heuristic": "mineurs",
        "sum-mineurs": True,
        "max-mineur-search-stop": 5,
        "simplification-tries": 1000,
        "num-samples": 10,
        "samples-folder": ’samples/calvin-pseudoquiral’,
    }
}
```

2.1.2. The output file

```
 Welcome to Listanalchem!
Remember to check the model you’re loading, it must:
* Have a name ‘modelname‘
* Have a list of species ‘species‘ (note: the first two species will be considered enantiomers,
  all other species do not)
* Have a non-empty list of reactions

******************************
***  Calvin - pseudoquiral  ***
******************************

Species:
[’L1’, ’D1’, ’L2’, ’D2’]
Reactions list:
[’L1 + L2 -> 2 L2’,
 ’D1 + D2 -> 2 D2’,
 ’2 L2 -> L1 + L2’,
 ’2 D2 -> D1 + D2’,
 ’L1 -> D1’,
 ’D1 -> L1’,
 ’L1 -> L2’,
 ’D1 -> D2’,
 ’L2 -> L1’,
 ’D2 -> D1’]
Dual pair equations (equations are numbered from 0 to 9):
[(0, 1), (2, 3), (6, 7), (8, 9)]
Regular reactions (not dual)
[4, 5]


Stoichiometric Matrix:
[[-1  0  1  0 -1  1 -1  0  1  0]
 [ 0 -1  0  1  1 -1  0 -1  0  1]
 [ 1  0 -1  0  0  0  1  0 -1  0]
 [ 0  1  0 -1  0  0  0  1  0 -1]]

Reactions Order Matrix:
[[1 0 0 0 1 0 1 0 0 0]
 [0 1 0 0 0 1 0 1 0 0]
 [1 0 2 0 0 0 0 0 1 0]
 [0 1 0 2 0 0 0 0 0 1]]

Velocity Function:
[k0*x0*x2]
[        ]
[k1*x1*x3]
[        ]
[      2 ]
[ k2*x2  ]
```

```
[         ]
[       2 ]
[ k3*x3   ]
[         ]
[ k4*x0   ]
[         ]
[ k5*x1   ]
[         ]
[ k6*x0   ]
[         ]
[ k7*x1   ]
[         ]
[ k8*x2   ]
[         ]
[ k9*x3   ]

Differential equations functions (polynomials) vector:
[                  2                              ]
[-k0*x0*x2 + k2*x2  - k4*x0 + k5*x1 - k6*x0 + k8*x2]
[                                                 ]
[                  2                              ]
[-k1*x1*x3 + k3*x3  + k4*x0 - k5*x1 - k7*x1 + k9*x3]
[                                                 ]
[                         2                       ]
[        k0*x0*x2 - k2*x2  + k6*x0 - k8*x2        ]
[                                                 ]
[                         2                       ]
[        k1*x1*x3 - k3*x3  + k7*x1 - k9*x3        ]

Warning: Stoichiometric Matrix is SINGULAR!


============================== ( - 2 - ) ==============================
*** Stoichiometric Network Analysis using the algorithm from reference [3] in README.md ***

Adding rows to the Stoichiometric Matrix encoding the dual pairs conditions in order to make
the cone defined by the Extreme Currents smaller and 'more precise'

Extended Stoichiometric matrix with rows indicating dual pairs
[[-1.  0.  1.  0. -1.  1. -1.  0.  1.  0.]
 [ 0. -1.  0.  1.  1. -1.  0. -1.  0.  1.]
 [ 1.  0. -1.  0.  0.  0.  1.  0. -1.  0.]
 [ 0.  1.  0. -1.  0.  0.  0.  1.  0. -1.]
 [ 1. -1.  0.  0.  0.  0.  0.  0.  0.  0.]
 [ 0.  0.  1. -1.  0.  0.  0.  0.  0.  0.]
 [ 0.  0.  0.  0.  0.  0.  1. -1.  0.  0.]
 [ 0.  0.  0.  0.  0.  0.  0.  0.  1. -1.]]

Extreme Currents Matrix (computed from Stoichiometric Matrix)
[[1 0 0 1 0]
```

```
 [1 0 0 1 0]
 [1 0 1 0 0]
 [1 0 1 0 0]
 [0 1 0 0 0]
 [0 1 0 0 0]
 [0 0 1 0 1]
 [0 0 1 0 1]
 [0 0 0 1 1]
 [0 0 0 1 1]]

E_omega_prim matrix
[j0 + j3]
[       ]
[j0 + j3]
[       ]
[j0 + j2]
[       ]
[j0 + j2]
[       ]
[  j1   ]
[       ]
[  j1   ]
[       ]
[j2 + j4]
[       ]
[j2 + j4]
[       ]
[j3 + j4]
[       ]
[j3 + j4]

V(J) Matrix
[-j0 - j1 - j2 - j3 - j4            j1             j0 + 2*j2 + j4          0
[
[          j1             -j0 - j1 - j2 - j3 - j4         0         j0 + 2*j2
[
[   j0 + j2 + j3 + j4                0             -j0 - 2*j2 - j4         0
[
[           0                j0 + j2 + j3 + j4            0         -j0 - 2*j2

     ]
     ]
+ j4 ]
     ]
     ]
     ]
 - j4]


---- Mineur Analysis ----
```

```
Limiting mineur analysis to mineurs of size smaller or equal to 5

There are no negative terms in the determinant of any mineur of size <= 5
According to this criteria the system seems to be stable.

============================= ( - 6 - ) ==============================

=== Algorithm based on the Frank inequality, according to reference [5] in README.md file ===

*** Solving semialgebraic problem (sampling from a non-linear inequalities indirectly) using
Clarke factorization according to the Stoichiometric Network Analysis (SNA) ***

Enantiomers divided into L's and D's groups
(('L1', 'L2'), ('D1', 'D2'))

Stoichiometric Matrix is SINGULAR!

Adding rows to the Stoichiometric Matrix encoding the dual pairs conditions in order to make
the cone defined by the Extreme Currents smaller and 'more precise'

Extended Stoichiometric matrix with rows indicating dual pairs
[[-1.  0.  1.  0. -1.  1. -1.  0.  1.  0.]
 [ 0. -1.  0.  1.  1. -1.  0. -1.  0.  1.]
 [ 1.  0. -1.  0.  0.  0.  1.  0. -1.  0.]
 [ 0.  1.  0. -1.  0.  0.  0.  1.  0. -1.]
 [ 1. -1.  0.  0.  0.  0.  0.  0.  0.  0.]
 [ 0.  0.  1. -1.  0.  0.  0.  0.  0.  0.]
 [ 0.  0.  0.  0.  0.  0.  1. -1.  0.  0.]
 [ 0.  0.  0.  0.  0.  0.  0.  0.  1. -1.]]

Extreme Currents Matrix (computed from Stoichiometric Matrix)
[[1 0 0 1 0]
 [1 0 0 1 0]
 [1 0 1 0 0]
 [1 0 1 0 0]
 [0 1 0 0 0]
 [0 1 0 0 0]
 [0 0 1 0 1]
 [0 0 1 0 1]
 [0 0 0 1 1]
 [0 0 0 1 1]]

E_omega column
[j0 + j3]
[       ]
[j0 + j3]
[       ]
[j0 + j2]
[       ]
[j0 + j2]
```

```
[       ]
[  j1   ]
[       ]
[  j1   ]
[       ]
[j2 + j4]
[       ]
[j2 + j4]
[       ]
[j3 + j4]
[       ]
[j3 + j4]

V(J) Matrix
[-j0 - j1 - j2 - j3 - j4           j1             j0 + 2*j2 + j4          0
[
[         j1             -j0 - j1 - j2 - j3 - j4         0         j0 + 2*j2
[
[   j0 + j2 + j3 + j4                0             -j0 - 2*j2 - j4         0
[
[          0                j0 + j2 + j3 + j4            0         -j0 - 2*j2

     ]
     ]
+ j4 ]
     ]
     ]
     ]
 - j4]

V(J) matrix reshaped to show clearly symetry [A B; B A] in Matrix
[-j0 - j1 - j2 - j3 - j4  j0 + 2*j2 + j4            j1                    0
[
[   j0 + j2 + j3 + j4     -j0 - 2*j2 - j4            0                    0
[
[         j1                     0         -j0 - j1 - j2 - j3 - j4  j0 + 2*j2
[
[          0                     0             j0 + j2 + j3 + j4     -j0 - 2*j2

     ]
     ]
     ]
     ]
+ j4 ]
     ]
 - j4]

'A' Matrix
[-j0 - j1 - j2 - j3 - j4  j0 + 2*j2 + j4 ]
[                                        ]
```

```
[   j0 + j2 + j3 + j4     -j0 - 2*j2 - j4]

'B' Matrix
[j1  0]
[     ]
[0   0]

New V(J) Matrix (A - B)
[-j0 - 2*j1 - j2 - j3 - j4  j0 + 2*j2 + j4 ]
[                                          ]
[    j0 + j2 + j3 + j4      -j0 - 2*j2 - j4]


---- Mineur Analysis ----
Limiting mineur analysis to mineurs of size smaller or equal to 5

There are no negative terms in the determinant of any mineur of size <= 5
According to this criteria the system seems to be stable.
******************************
***  Calvin - pseudoquiral  ***
******************************
```

2.2. The replicator model of Hochberg and Ribo

2.2.1. The input file

```
 # run with:
# > python -m listanalchem --model models/Replicator-HR-equals.py

modelname = 'Replicator Hochberg-Ribo-Equals'
species = ['R1D', 'R1L', 'R2D', 'R2L', 'A']
reactions = [
    'A + R1D + R2D -> 2 R1D + R2D',  # 0
    '2 R1D + R2D -> A + R1D + R2D',  # 1
    'A + R1L + R2L -> 2 R1L + R2L',  # 2
    '2 R1L + R2L -> A + R1L + R2L',  # 3
    'A + R1D + R2D -> R1D + 2 R2D',  # 4
    'R1D + 2 R2D -> A + R1D + R2D',  # 5
    'A + R1L + R2L -> R1L + 2 R2L',  # 6
    'R1L + 2 R2L -> A + R1L + R2L',  # 7
    'R1D ->',  # 8
    'R2D ->',  # 9
    'R1L ->',  # 10
    'R2L ->',  # 11
    '-> A',    # 12
    'A ->'     # 13
]
dual_pairs = [(0, 2), (1, 3), (4, 6), (5, 7), (8, 9), (9, 10), (10, 11),(11,13),(2,4),(3,5)]

analyses = {
    "trace-determinant": {
```

```
            "enabled": False,
            "2by2-jacobian": True
        },
        "sna": {
            "enabled": False,
            "dual-pairs-in-ec": True,
            # "instability-heuristic": "trace-determinant",
            # "instability-heuristic": "characteristic-polynomial",
            "instability-heuristic": "mineurs",
            "sum-mineurs": True,
            "max-mineur-search-stop": 5,
            "simplification-tries": 1000,
            "num-samples": 10,
            "samples-folder": 'samples/replicator',
        },
        "six-categories": {
            "enabled": False,
            "num-samples": 10,
            "samples-folder": 'samples/iwamoto_imperfect',
        },
        "frank-ineq-nonlinear": {
            "enabled": False
        },
        "frank-ineq-linear": {
            "enabled": False,
            "dual-pairs-in-ec": True,
            "num-samples": 1,
            "samples-folder": None,
            "samples-for-proportion": 1000
        },
        "frank-pseudoquiral": {
            "enabled": True,
            "enantiomeric-pairs": [(0, 1), (2, 3)],
            "dual-pairs-in-ec": True,
            # "instability-heuristic": "trace-determinant",
            # "instability-heuristic": "characteristic-polynomial",
            "instability-heuristic": "mineurs",
            "sum-mineurs": True,
            "max-mineur-search-stop": 5,
            "simplification-tries": 12000,
            "num-samples": 10,
            "samples-folder": 'samples/replicator',
        }
}
```

2.2.2. The output file

```
 Welcome to Listanalchem!
Remember to check the model you're loading, it must:
* Have a name `modelname`
* Have a list of species `species` (note: the first two species will be considered
```

```
enantiomers, all other species do not)
* Have a non-empty list of reactions

****************************************
***  Replicator Hochberg-Ribo-Equals  ***
****************************************

Species:
['R1D', 'R1L', 'R2D', 'R2L', 'A']
Reactions list:
['R1D + R2D + A -> 2 R1D + R2D',
 '2 R1D + R2D -> R1D + R2D + A',
 'R1L + R2L + A -> 2 R1L + R2L',
 '2 R1L + R2L -> R1L + R2L + A',
 'R1D + R2D + A -> R1D + 2 R2D',
 'R1D + 2 R2D -> R1D + R2D + A',
 'R1L + R2L + A -> R1L + 2 R2L',
 'R1L + 2 R2L -> R1L + R2L + A',
 'R1D -> ',
 'R2D -> ',
 'R1L -> ',
 'R2L -> ',
 ' -> A',
 'A -> ']
Dual pair equations (equations are numbered from 0 to 13):
[(0, 2),
 (1, 3),
 (4, 6),
 (5, 7),
 (8, 9),
 (9, 10),
 (10, 11),
 (11, 13),
 (2, 4),
 (3, 5)]
Regular reactions (not dual)
[12]


Stoichiometric Matrix:
[[ 1 -1  0  0  0  0  0  0 -1  0  0  0  0  0]
 [ 0  0  1 -1  0  0  0  0  0  0 -1  0  0  0]
 [ 0  0  0  0  1 -1  0  0  0 -1  0  0  0  0]
 [ 0  0  0  0  0  0  1 -1  0  0  0 -1  0  0]
 [-1  1 -1  1 -1  1 -1  1  0  0  0  0  1 -1]]

Reactions Order Matrix:
[[1 2 0 0 1 1 0 0 1 0 0 0 0 0]
 [0 0 1 2 0 0 1 1 0 0 1 0 0 0]
 [1 1 0 0 1 2 0 0 0 1 0 0 0 0]
```

```
 [0 0 1 1 0 0 1 2 0 0 0 1 0 0]
 [1 0 1 0 1 0 1 0 0 0 0 0 0 1]]

Velocity Function:
[k0*x0*x2*x4]
[           ]
[      2    ]
[ k1*x0 *x2 ]
[           ]
[k2*x1*x3*x4]
[           ]
[      2    ]
[ k3*x1 *x3 ]
[           ]
[k4*x0*x2*x4]
[           ]
[         2 ]
[ k5*x0*x2  ]
[           ]
[k6*x1*x3*x4]
[           ]
[         2 ]
[ k7*x1*x3  ]
[           ]
[   k8*x0   ]
[           ]
[   k9*x2   ]
[           ]
[  k10*x1   ]
[           ]
[  k11*x3   ]
[           ]
[    k12    ]
[           ]
[  k13*x4   ]

Differential equations functions (polynomials) vector:
[                                                              2
[                                           k0*x0*x2*x4 - k1*x0 *x2 - k8*x0
[
[                                                                       2
[                                          -k10*x1 + k2*x1*x3*x4 - k3*x1 *x3
[
[                                                                 2
[                                           k4*x0*x2*x4 - k5*x0*x2  - k9*x2
[
[                                                                          2
[                                          -k11*x3 + k6*x1*x3*x4 - k7*x1*x3
[
[                    2                                         2
```

```
[-k0*x0*x2*x4 + k1*x0 *x2 + k12 - k13*x4 - k2*x1*x3*x4 + k3*x1 *x3 - k4*x0*x2*

                                        ]
                                        ]
                                        ]
                                        ]
                                        ]
                                        ]
                                        ]
                                        ]
                                        ]
                                        ]
                                        ]
                                        ]
             2                         2]
x4 + k5*x0*x2  - k6*x1*x3*x4 + k7*x1*x3 ]


============================= ( - 6 - ) =============================

=== Algorithm based on the Frank inequality, according to reference [5] in README.md file ===

*** Solving semialgebraic problem (sampling from a non-linear inequalities indirectly)
using Clarke factorization according to the Stoichiometric Network Analysis (SNA) ***

Enantiomers divided into L's and D's groups
(('R1D', 'R2D'), ('R1L', 'R2L'))

Adding rows to the Stoichiometric Matrix encoding the dual pairs conditions in order to
make the cone defined by the Extreme Currents smaller and 'more precise'

Extended Stoichiometric matrix with rows indicating dual pairs
[[ 1. -1.  0.  0.  0.  0.  0.  0. -1.  0.  0.  0.  0.  0.]
 [ 0.  0.  1. -1.  0.  0.  0.  0.  0.  0. -1.  0.  0.  0.]
 [ 0.  0.  0.  0.  1. -1.  0.  0.  0. -1.  0.  0.  0.  0.]
 [ 0.  0.  0.  0.  0.  0.  1. -1.  0.  0.  0. -1.  0.  0.]
 [-1.  1. -1.  1. -1.  1. -1.  1.  0.  0.  0.  0.  1. -1.]
 [ 1.  0. -1.  0.  0.  0.  0.  0.  0.  0.  0.  0.  0.  0.]
 [ 0.  1.  0. -1.  0.  0.  0.  0.  0.  0.  0.  0.  0.  0.]
 [ 0.  0.  0.  0.  1.  0. -1.  0.  0.  0.  0.  0.  0.  0.]
 [ 0.  0.  0.  0.  0.  1.  0. -1.  0.  0.  0.  0.  0.  0.]
 [ 0.  0.  0.  0.  0.  0.  0.  0.  1. -1.  0.  0.  0.  0.]
 [ 0.  0.  0.  0.  0.  0.  0.  0.  0.  1. -1.  0.  0.  0.]
 [ 0.  0.  0.  0.  0.  0.  0.  0.  0.  0.  1. -1.  0.  0.]
 [ 0.  0.  0.  0.  0.  0.  0.  0.  0.  0.  0.  1.  0. -1.]
 [ 0.  0.  1.  0. -1.  0.  0.  0.  0.  0.  0.  0.  0.  0.]
 [ 0.  0.  0.  1.  0. -1.  0.  0.  0.  0.  0.  0.  0.  0.]]

Extreme Currents Matrix (computed from Stoichiometric Matrix)
[[1 1]
```

```
 [1 0]
 [1 1]
 [1 0]
 [1 1]
 [1 0]
 [1 1]
 [1 0]
 [0 1]
 [0 1]
 [0 1]
 [0 1]
 [0 5]
 [0 1]]

E_omega column
[j0 + j1]
[       ]
[  j0   ]
[       ]
[j0 + j1]
[       ]
[  j0   ]
[       ]
[j0 + j1]
[       ]
[  j0   ]
[       ]
[j0 + j1]
[       ]
[  j0   ]
[       ]
[  j1   ]
[       ]
[  j1   ]
[       ]
[  j1   ]
[       ]
[  j1   ]
[       ]
[ 5*j1  ]
[       ]
[  j1   ]

V(J) Matrix
[   -j0         0         j1          0        j0 + j1   ]
[                                                         ]
[    0         -j0          0         j1        j0 + j1   ]
[                                                         ]
[   j1          0         -j0          0        j0 + j1   ]
[                                                         ]
```

```
[    0         j1          0         -j0       j0 + j1   ]
[                                                         ]
[j0 - 2*j1  j0 - 2*j1  j0 - 2*j1  j0 - 2*j1  -4*j0 - 5*j1]

V(J) matrix reshaped to show clearly symetry [A B; B A] in Matrix
[   -j0        j1          0          0        j0 + j1   ]
[                                                         ]
[   j1         -j0         0          0        j0 + j1   ]
[                                                         ]
[    0          0         -j0        j1        j0 + j1   ]
[                                                         ]
[    0          0         j1         -j0       j0 + j1   ]
[                                                         ]
[j0 - 2*j1  j0 - 2*j1  j0 - 2*j1  j0 - 2*j1  -4*j0 - 5*j1]

'A' Matrix
[-j0  j1 ]
[        ]
[j1   -j0]

'B' Matrix
[0  0]
[    ]
[0  0]

New V(J) Matrix (A - B)
[-j0  j1 ]
[        ]
[j1   -j0]


---- Mineur Analysis ----
Limiting mineur analysis to mineurs of size smaller or equal to 5

Mineur:
Poly(j0**2 - j1**2, j0, j1, domain='ZZ')
Compounds number: [(0, 1)]

---- --------------- ----
Restrictions to solve
[j0 > 0, j1 > 0, -j0**2 + j1**2 > 0]

Trying to find a solution with the restrictions

Found a solution on try number 1 of 12000

(Randomly) Chosen variable values:
[j1: 0.814798351563,
 ]
```

```
Values that variables can take given the already chosen (see above) values for variables [j1]
[j0 = Interval.open(0, 0.814798000000000)
 ]

Sampling 10 points from js intervals
Saving sampled models to ‘samples/replicator‘
****************************************
***  Replicator Hochberg-Ribo-Equals  ***
****************************************
```

2.3. The APED model of Plasson et. al

2.3.1. The input file

```
 # APED model 2004. Plasson-Bersini-Commeyras.

# run with "python -m listanalchem --model models/APED-2004.py"

modelname = 'APED'
species = ['L', 'La', 'D', 'Da', 'LL', 'DL', 'LD', 'DD']
reactions = [
    'L -> La',       # 0  - ka
    'D -> Da',       # 1  - ka
    'La -> L',       # 2  - kb
    'Da -> D',       # 3  - kb
    'La + L -> LL',  # 4  - kp
    'Da + L -> DL',  # 5  - kalfap
    'La + D -> LD',  # 6  - kalfap
    'Da + D -> DD',  # 7  - kp
    'LL -> L + L',   # 8  - kh
    'DL -> L + D',   # 9  - kbetah
    'LD -> L + D',   # 10 - kbetah
    'DD -> D + D',   # 11 - kh
    'LD -> DD',      # 12 - ke
    'DD -> LD',      # 13 - kgammae
    'LL -> DL',      # 14 - kgammae
    'DL -> LL'       # 15 - ke
]

dual_pairs = [(0,1),(2,3),(4,7),(5,6),(8,11),(9,10),(12,15),(13,14)]

analyses = {
    "sna": {
        "enabled": False,
        "dual-pairs-in-ec": True,
        # "instability-heuristic": "trace-determinant",
        # "instability-heuristic": "characteristic-polynomial",
        "instability-heuristic": "mineurs",
        "sum-mineurs": True,
        "max-mineur-search-stop": 5,
        "simplification-tries": 10000,
```

```
        "num-samples": 10,
        "samples-folder": 'samples/aped_2004',
    },
    "frank-pseudoquiral": {
        "enabled": True,
        "enantiomeric-pairs": [(0, 2), (1, 3), (4, 7), (5, 6)],
        "dual-pairs-in-ec": True,
        # "instability-heuristic": "trace-determinant",
        # "instability-heuristic": "characteristic-polynomial",
        "instability-heuristic": "mineurs",
        "sum-mineurs": True,
        "max-mineur-search-stop": 5,
        "simplification-tries": 10000,
        "num-samples": 10,
        "samples-folder": 'samples/aped_2004',
    }
}
```

2.3.2. The output file

```
 Welcome to Listanalchem!
Remember to check the model you're loading, it must:
* Have a name `modelname`
* Have a list of species `species` (note: the first two species will be considered
  enantiomers, all other species do not)
* Have a non-empty list of reactions

**************
***  APED  ***
**************

Species:
['L', 'La', 'D', 'Da', 'LL', 'DL', 'LD', 'DD']
Reactions list:
['L -> La',
 'D -> Da',
 'La -> L',
 'Da -> D',
 'L + La -> LL',
 'L + Da -> DL',
 'La + D -> LD',
 'D + Da -> DD',
 'LL -> 2 L',
 'DL -> L + D',
 'LD -> L + D',
 'DD -> 2 D',
 'LD -> DD',
 'DD -> LD',
 'LL -> DL',
 'DL -> LL']
Dual pair equations (equations are numbered from 0 to 15):
```

```
[(0, 1), (2, 3), (4, 7), (5, 6), (8, 11), (9, 10), (12, 15), (13, 14)]

Stoichiometric Matrix:
[[-1  0  1  0 -1 -1  0  0  2  1  1  0  0  0  0  0]
 [ 1  0 -1  0 -1  0 -1  0  0  0  0  0  0  0  0  0]
 [ 0 -1  0  1  0  0 -1 -1  0  1  1  2  0  0  0  0]
 [ 0  1  0 -1  0 -1  0 -1  0  0  0  0  0  0  0  0]
 [ 0  0  0  0  1  0  0  0 -1  0  0  0  0  0 -1  1]
 [ 0  0  0  0  0  1  0  0  0 -1  0  0  0  0  1 -1]
 [ 0  0  0  0  0  0  1  0  0  0 -1  0 -1  1  0  0]
 [ 0  0  0  0  0  0  0  1  0  0  0 -1  1 -1  0  0]]

Reactions Order Matrix:
[[1 0 0 0 1 1 0 0 0 0 0 0 0 0 0 0]
 [0 0 1 0 1 0 1 0 0 0 0 0 0 0 0 0]
 [0 1 0 0 0 0 1 1 0 0 0 0 0 0 0 0]
 [0 0 0 1 0 1 0 1 0 0 0 0 0 0 0 0]
 [0 0 0 0 0 0 0 0 1 0 0 0 0 0 1 0]
 [0 0 0 0 0 0 0 0 0 1 0 0 0 0 0 1]
 [0 0 0 0 0 0 0 0 0 0 1 0 1 0 0 0]
 [0 0 0 0 0 0 0 0 0 0 0 1 0 1 0 0]]

Velocity Function:
[ k0*x0  ]
[        ]
[ k1*x2  ]
[        ]
[ k2*x1  ]
[        ]
[ k3*x3  ]
[        ]
[k4*x0*x1]
[        ]
[k5*x0*x3]
[        ]
[k6*x1*x2]
[        ]
[k7*x2*x3]
[        ]
[ k8*x4  ]
[        ]
[ k9*x5  ]
[        ]
[ k10*x6 ]
[        ]
[ k11*x7 ]
[        ]
[ k12*x6 ]
[        ]
[ k13*x7 ]
```

```
[        ]
[ k14*x4 ]
[        ]
[ k15*x5 ]

Differential equations functions (polynomials) vector:
[-k0*x0 + k10*x6 + k2*x1 - k4*x0*x1 - k5*x0*x3 + 2*k8*x4 + k9*x5 ]
[                                                                 ]
[              k0*x0 - k2*x1 - k4*x0*x1 - k6*x1*x2                ]
[                                                                 ]
[-k1*x2 + k10*x6 + 2*k11*x7 + k3*x3 - k6*x1*x2 - k7*x2*x3 + k9*x5]
[                                                                 ]
[              k1*x2 - k3*x3 - k5*x0*x3 - k7*x2*x3                ]
[                                                                 ]
[              -k14*x4 + k15*x5 + k4*x0*x1 - k8*x4                ]
[                                                                 ]
[               k14*x4 - k15*x5 + k5*x0*x3 - k9*x5                ]
[                                                                 ]
[              -k10*x6 - k12*x6 + k13*x7 + k6*x1*x2               ]
[                                                                 ]
[              -k11*x7 + k12*x6 - k13*x7 + k7*x2*x3               ]

Warning: Stoichiometric Matrix is SINGULAR!


============================= ( - 6 - ) =============================

=== Algorithm based on the Frank inequality, according to reference [5] in README.md file ===

*** Solving semialgebraic problem (sampling from a non-linear inequalities indirectly)
using Clarke factorization according to the Stoichiometric Network Analysis (SNA) ***

Enantiomers divided into L's and D's groups
(('L', 'La', 'LL', 'DL'), ('D', 'Da', 'DD', 'LD'))

Stoichiometric Matrix is SINGULAR!

Adding rows to the Stoichiometric Matrix encoding the dual pairs conditions in order to
make the cone defined by the Extreme Currents smaller and 'more precise'

Extended Stoichiometric matrix with rows indicating dual pairs
[[-1.  0.  1.  0. -1. -1.  0.  0.  2.  1.  1.  0.  0.  0.  0.  0.]
 [ 1.  0. -1.  0. -1.  0. -1.  0.  0.  0.  0.  0.  0.  0.  0.  0.]
 [ 0. -1.  0.  1.  0.  0. -1. -1.  0.  1.  1.  2.  0.  0.  0.  0.]
 [ 0.  1.  0. -1.  0. -1.  0. -1.  0.  0.  0.  0.  0.  0.  0.  0.]
 [ 0.  0.  0.  0.  1.  0.  0.  0. -1.  0.  0.  0.  0.  0. -1.  1.]
 [ 0.  0.  0.  0.  0.  1.  0.  0.  0. -1.  0.  0.  0.  0.  1. -1.]
 [ 0.  0.  0.  0.  0.  0.  1.  0.  0.  0. -1.  0. -1.  1.  0.  0.]
 [ 0.  0.  0.  0.  0.  0.  0.  1.  0.  0.  0. -1.  1. -1.  0.  0.]
 [ 1. -1.  0.  0.  0.  0.  0.  0.  0.  0.  0.  0.  0.  0.  0.  0.]
```

```
 [ 0.  0.  1. -1.  0.  0.  0.  0.  0.  0.  0.  0.  0.  0.  0.  0.]
 [ 0.  0.  0.  0.  1.  0.  0. -1.  0.  0.  0.  0.  0.  0.  0.  0.]
 [ 0.  0.  0.  0.  0.  1. -1.  0.  0.  0.  0.  0.  0.  0.  0.  0.]
 [ 0.  0.  0.  0.  0.  0.  0.  0.  1.  0.  0. -1.  0.  0.  0.  0.]
 [ 0.  0.  0.  0.  0.  0.  0.  0.  0.  1. -1.  0.  0.  0.  0.  0.]
 [ 0.  0.  0.  0.  0.  0.  0.  0.  0.  0.  0.  0.  1.  0.  0. -1.]
 [ 0.  0.  0.  0.  0.  0.  0.  0.  0.  0.  0.  0.  0.  1. -1.  0.]]

Extreme Currents Matrix (computed from Stoichiometric Matrix)
[[1 1 1 1 1 0]
 [1 1 1 1 1 0]
 [1 0 0 0 0 0]
 [1 0 0 0 0 0]
 [0 0 1 1 0 0]
 [0 1 0 0 1 0]
 [0 1 0 0 1 0]
 [0 0 1 1 0 0]
 [0 0 1 0 1 0]
 [0 1 0 1 0 0]
 [0 1 0 1 0 0]
 [0 0 1 0 1 0]
 [0 0 0 0 1 1]
 [0 0 0 1 0 1]
 [0 0 0 1 0 1]
 [0 0 0 0 1 1]]

E_omega column
[j0 + j1 + j2 + j3 + j4]
[                      ]
[j0 + j1 + j2 + j3 + j4]
[                      ]
[          j0          ]
[                      ]
[          j0          ]
[                      ]
[       j2 + j3        ]
[                      ]
[       j1 + j4        ]
[                      ]
[       j1 + j4        ]
[                      ]
[       j2 + j3        ]
[                      ]
[       j2 + j4        ]
[                      ]
[       j1 + j3        ]
[                      ]
[       j1 + j3        ]
[                      ]
[       j2 + j4        ]
```

```
[                      ]
[       j4 + j5        ]
[                      ]
[       j3 + j5        ]
[                      ]
[       j3 + j5        ]
[                      ]
[       j4 + j5        ]

V(J) Matrix
[-j0 - 2*j1 - 2*j2 - 2*j3 - 2*j4       j0 - j2 - j3                  0
[
[         j0 + j1 + j4            -j0 - j1 - j2 - j3 - j4            -j1 - j4
[
[               0                         -j1 - j4         -j0 - 2*j1 - 2*j2 -
[
[           -j1 - j4                          0                   j0 + j1 +
[
[            j2 + j3                       j2 + j3                   0
[
[            j1 + j4                          0                      0
[
[               0                          j1 + j4                j1 + j4
[
[               0                             0                   j2 + j3

                     -j1 - j4            2*j2 + 2*j4           j1 + j3

                         0                    0                   0

 2*j3 - 2*j4       j0 - j2 - j3               0               j1 + j3

j4            -j0 - j1 - j2 - j3 - j4         0                   0

                         0           -j2 - j3 - j4 - j5       j4 + j5

                      j1 + j4              j3 + j5        -j1 - j3 - j4 - j5

                         0                    0                   0

                      j2 + j3                 0                   0

      j1 + j3                 0          ]
                                         ]
         0                    0          ]
                                         ]
      j1 + j3            2*j2 + 2*j4     ]
                                         ]
         0                    0          ]
                                         ]
```

```
         0                   0         ]
                                       ]
         0                   0         ]
                                       ]
 -j1 - j3 - j4 - j5       j3 + j5      ]
                                       ]
      j4 + j5        -j2 - j3 - j4 - j5]

V(J) matrix reshaped to show clearly symetry [A B; B A] in Matrix
[-j0 - 2*j1 - 2*j2 - 2*j3 - 2*j4      j0 - j2 - j3          2*j2 + 2*j4
[
[         j0 + j1 + j4           -j0 - j1 - j2 - j3 - j4          0
[
[            j2 + j3                      j2 + j3          -j2 - j3 - j4 - j5
[
[            j1 + j4                         0                  j3 + j5
[
[               0                         -j1 - j4                0
[
[           -j1 - j4                         0                    0
[
[               0                            0                    0
[
[               0                          j1 + j4                0

      j1 + j3                  0                        -j1 - j4

         0                  -j1 - j4                        0

      j4 + j5                  0                            0

 -j1 - j3 - j4 - j5            0                          j1 + j4

      j1 + j3        -j0 - 2*j1 - 2*j2 - 2*j3 - 2*j4      j0 - j2 - j3

         0                 j0 + j1 + j4           -j0 - j1 - j2 - j3 - j4

         0                    j2 + j3                     j2 + j3

         0                    j1 + j4                       0

         0                j1 + j3      ]
                                       ]
         0                   0         ]
                                       ]
         0                   0         ]
                                       ]
         0                   0         ]
                                       ]
    2*j2 + 2*j4           j1 + j3      ]
```

```
                                         ]
        0                   0            ]
                                         ]
 -j2 - j3 - j4 - j5       j4 + j5        ]
                                         ]
     j3 + j5        -j1 - j3 - j4 - j5]

'A' Matrix
[-j0 - 2*j1 - 2*j2 - 2*j3 - 2*j4       j0 - j2 - j3           2*j2 + 2*j4
[
[         j0 + j1 + j4            -j0 - j1 - j2 - j3 - j4          0
[
[            j2 + j3                          j2 + j3          -j2 - j3 - j4 - j5
[
[            j1 + j4                            0                   j3 + j5

     j1 + j3      ]
                  ]
        0         ]
                  ]
     j4 + j5      ]
                  ]
 -j1 - j3 - j4 - j5]

'B' Matrix
[   0      -j1 - j4  0  j1 + j3]
[                              ]
[-j1 - j4     0      0     0   ]
[                              ]
[   0         0      0     0   ]
[                              ]
[   0      j1 + j4   0     0   ]

New V(J) Matrix (A - B)
[-j0 - 2*j1 - 2*j2 - 2*j3 - 2*j4  j0 + j1 - j2 - j3 + j4      2*j2 + 2*j4
[
[       j0 + 2*j1 + 2*j4          -j0 - j1 - j2 - j3 - j4          0
[
[            j2 + j3                          j2 + j3          -j2 - j3 - j4 - j5
[
[            j1 + j4                          -j1 - j4              j3 + j5

        0         ]
                  ]
        0         ]
                  ]
     j4 + j5      ]
                  ]
 -j1 - j3 - j4 - j5]
```

```
---- Mineur Analysis ----
Limiting mineur analysis to mineurs of size smaller or equal to 5

Mineur:
Poly(4*j0*j1*j2*j3 + 4*j0*j1*j2*j5 + 4*j0*j1*j3**2 + 4*j0*j1*j3*j5 + 4*j0*j2*j
3**2 + 4*j0*j2*j3*j5 + 4*j0*j3**3 + 4*j0*j3**2*j5 + 6*j1**2*j2*j3 + 2*j1**2*j2
*j4 + 8*j1**2*j2*j5 + 6*j1**2*j3**2 + 6*j1**2*j3*j5 + 2*j1**2*j4**2 + 2*j1**2*
j4*j5 + 2*j1*j2**2*j3 - 2*j1*j2**2*j4 + 10*j1*j2*j3**2 + 4*j1*j2*j3*j4 + 8*j1*
j2*j3*j5 + 2*j1*j2*j4**2 + 8*j1*j2*j4*j5 + 8*j1*j3**3 + 6*j1*j3**2*j4 + 8*j1*j
3**2*j5 - 2*j1*j3*j4**2 + 4*j1*j3*j4*j5 + 4*j1*j4**3 + 4*j1*j4**2*j5 + 2*j2**2
*j3**2 + 2*j2**2*j3*j5 - 2*j2**2*j4**2 - 2*j2**2*j4*j5 + 4*j2*j3**3 + 6*j2*j3*
*2*j4 + 4*j2*j3**2*j5 - 2*j2*j3*j4**2 + 4*j2*j3*j4*j5 + 2*j3**4 + 6*j3**3*j4 +
 2*j3**3*j5 + 6*j3**2*j4*j5 - 2*j3*j4**3 - 2*j3*j4**2*j5 + 2*j4**4 + 2*j4**3*j
5, j0, j1, j2, j3, j4, j5, domain='ZZ')
Compounds number: [(0, 1, 2, 3)]

---- -------------- ----
Restrictions to solve
[j0 > 0,
 j1 > 0,
 j2 > 0,
 j3 > 0,
 j4 > 0,
 j5 > 0,
 -4*j0*j1*j2*j3 - 4*j0*j1*j2*j5 - 4*j0*j1*j3**2 - 4*j0*j1*j3*j5 -
 4*j0*j2*j3**2 - 4*j0*j2*j3*j5 - 4*j0*j3**3 - 4*j0*j3**2*j5 -
 6*j1**2*j2*j3 - 2*j1**2*j2*j4 - 8*j1**2*j2*j5 - 6*j1**2*j3**2 -
 6*j1**2*j3*j5 - 2*j1**2*j4**2 - 2*j1**2*j4*j5 - 2*j1*j2**2*j3 +
 2*j1*j2**2*j4 - 10*j1*j2*j3**2 - 4*j1*j2*j3*j4 - 8*j1*j2*j3*j5 -
 2*j1*j2*j4**2 - 8*j1*j2*j4*j5 - 8*j1*j3**3 - 6*j1*j3**2*j4 -
 8*j1*j3**2*j5 + 2*j1*j3*j4**2 - 4*j1*j3*j4*j5 - 4*j1*j4**3 -
 4*j1*j4**2*j5 - 2*j2**2*j3**2 - 2*j2**2*j3*j5 + 2*j2**2*j4**2 +
 2*j2**2*j4*j5 - 4*j2*j3**3 - 6*j2*j3**2*j4 - 4*j2*j3**2*j5 +
 2*j2*j3*j4**2 - 4*j2*j3*j4*j5 - 2*j3**4 - 6*j3**3*j4 - 2*j3**3*j5 -
 6*j3**2*j4*j5 + 2*j3*j4**3 + 2*j3*j4**2*j5 - 2*j4**4 - 2*j4**3*j5 > 0]

Trying to find a solution with the restrictions

Found a solution on try number 33 of 10000

(Randomly) Chosen variable values:
[j3: 0.101800290882,
 j0: 0.529336072892,
 j2: 1.92129144847,
 j4: 1.51730047593,
 j1: 0.382895906423,
 ]

Values that variables can take given the already chosen (see above) values for
```

```
variables [j3, j4, j1, j2, j0]
[j5 = (0, 683403118501363/15617688553613300)]

Sampling 10 points from js intervals
Saving sampled models to ‘samples/aped_2004‘
**************
***  APED  ***
**************
```

2.4. The Iwamoto model

2.4.1. The Iwamoto model under perfect conditions

The input file

```
# run with "python -m listanalchem --model models/Iwamoto-Perfect.py"
modelname = 'Iwamoto-Perfect'
species = ["L", "D", "E-L", "E-D", "A"]
reactions = [
    "<-> A",          # 0 & 1.
    "A + L <-> 2L",   # 2 & 3.
    "A + D <-> 2D",   # 4 & 5.
    "L + E-L <->",    # 6 & 7.
    "D + E-D <->",    # 8 & 9.
    "-> E-L",         # 10.
    "-> E-D"          # 11.
]
dual_pairs = [(2, 4), (3, 5), (6, 8), (7, 9), (10, 11)]

analyses = {
    "frank-pseudoquiral": {
        "enabled": True,
        "enantiomeric-pairs": [(0, 1), (2, 3)],
        "dual-pairs-in-ec": True,
        # "instability-heuristic": "trace-determinant",
        # "instability-heuristic": "characteristic-polynomial",
        "instability-heuristic": "mineurs",
        "sum-mineurs": True,
        "max-mineur-search-stop": 5,
        "simplification-tries": 1000,
        "num-samples": 10,
        "samples-folder": 'samples/iwamoto_perfect',
    }
}
```

The output file

```
 Welcome to Listanalchem!
Remember to check the model you're loading, it must:
* Have a name ‘modelname‘
* Have a list of species ‘species‘ (note: the first two species will be considered
  enantiomers, all other species do not)
```

```
* Have a non-empty list of reactions

*************************
***  Iwamoto-Perfect  ***
*************************

Species:
['L', 'D', 'E-L', 'E-D', 'A']
Reactions list:
[' -> A',
 'A -> ',
 'L + A -> 2 L',
 '2 L -> L + A',
 'D + A -> 2 D',
 '2 D -> D + A',
 'L + E-L -> ',
 ' -> L + E-L',
 'D + E-D -> ',
 ' -> D + E-D',
 ' -> E-L',
 ' -> E-D']
Dual pair equations (equations are numbered from 0 to 11):
[(2, 4), (3, 5), (6, 8), (7, 9), (10, 11)]
Regular reactions (not dual)
[0, 1]


Stoichiometric Matrix:
[[ 0  0  1 -1  0  0 -1  1  0  0  0  0]
 [ 0  0  0  0  1 -1  0  0 -1  1  0  0]
 [ 0  0  0  0  0  0 -1  1  0  0  1  0]
 [ 0  0  0  0  0  0  0  0 -1  1  0  1]
 [ 1 -1 -1  1 -1  1  0  0  0  0  0  0]]

Reactions Order Matrix:
[[0 0 1 2 0 0 1 0 0 0 0 0]
 [0 0 0 0 1 2 0 0 1 0 0 0]
 [0 0 0 0 0 0 1 0 0 0 0 0]
 [0 0 0 0 0 0 0 0 1 0 0 0]
 [0 1 1 0 1 0 0 0 0 0 0 0]]

Velocity Function:
[   k0   ]
[        ]
[ k1*x4  ]
[        ]
[k2*x0*x4]
[        ]
[      2 ]
[ k3*x0  ]
```

```
[         ]
[k4*x1*x4]
[         ]
[       2 ]
[ k5*x1   ]
[         ]
[k6*x0*x2]
[         ]
[   k7    ]
[         ]
[k8*x1*x3]
[         ]
[   k9    ]
[         ]
[  k10    ]
[         ]
[  k11    ]

Differential equations functions (polynomials) vector:
[                        2                         ]
[        k2*x0*x4 - k3*x0  - k6*x0*x2 + k7         ]
[                                                  ]
[                        2                         ]
[        k4*x1*x4 - k5*x1  - k8*x1*x3 + k9         ]
[                                                  ]
[               k10 - k6*x0*x2 + k7                ]
[                                                  ]
[               k11 - k8*x1*x3 + k9                ]
[                                                  ]
[                             2                   2]
[k0 - k1*x4 - k2*x0*x4 + k3*x0  - k4*x1*x4 + k5*x1 ]


============================== ( - 6 - ) ==============================

=== Algorithm based on the Frank inequality, according to reference [5] in README.md file ===

*** Solving semialgebraic problem (sampling from a non-linear inequalities indirectly) using
    Clarke factorization according to the Stoichiometric Network Analysis (SNA) ***

Enantiomers divided into L's and D's groups
(('L', 'E-L'), ('D', 'E-D'))

Adding rows to the Stoichiometric Matrix encoding the dual pairs conditions in order to make
the cone defined by the Extreme Currents smaller and 'more precise'

Extended Stoichiometric matrix with rows indicating dual pairs
[[ 0.  0.  1. -1.  0.  0. -1.  1.  0.  0.  0.  0.]
 [ 0.  0.  0.  0.  1. -1.  0.  0. -1.  1.  0.  0.]
 [ 0.  0.  0.  0.  0.  0. -1.  1.  0.  0.  1.  0.]
```

```
 [ 0.  0.  0.  0.  0.  0.  0.  0. -1.  1.  0.  1.]
 [ 1. -1. -1.  1. -1.  1.  0.  0.  0.  0.  0.  0.]
 [ 0.  0.  1.  0. -1.  0.  0.  0.  0.  0.  0.  0.]
 [ 0.  0.  0.  1.  0. -1.  0.  0.  0.  0.  0.  0.]
 [ 0.  0.  0.  0.  0.  0.  1.  0. -1.  0.  0.  0.]
 [ 0.  0.  0.  0.  0.  0.  0.  1.  0. -1.  0.  0.]
 [ 0.  0.  0.  0.  0.  0.  0.  0.  0.  0.  1. -1.]]

Extreme Currents Matrix (computed from Stoichiometric Matrix)
[[1 0 0 2]
 [1 0 0 0]
 [0 1 0 1]
 [0 1 0 0]
 [0 1 0 1]
 [0 1 0 0]
 [0 0 1 1]
 [0 0 1 0]
 [0 0 1 1]
 [0 0 1 0]
 [0 0 0 1]
 [0 0 0 1]]

E_omega column
[j0 + 2*j3]
[         ]
[   j0    ]
[         ]
[ j1 + j3 ]
[         ]
[   j1    ]
[         ]
[ j1 + j3 ]
[         ]
[   j1    ]
[         ]
[ j2 + j3 ]
[         ]
[   j2    ]
[         ]
[ j2 + j3 ]
[         ]
[   j2    ]
[         ]
[   j3    ]
[         ]
[   j3    ]

V(J) Matrix
[-j1 - j2     0      -j2 - j3     0            j1 + j3     ]
[                                                          ]
```

```
[   0      -j1 - j2     0      -j2 - j3       j1 + j3     ]
[                                                         ]
[-j2 - j3     0      -j2 - j3     0             0         ]
[                                                         ]
[   0      -j2 - j3     0      -j2 - j3         0         ]
[                                                         ]
[j1 - j3   j1 - j3      0         0      -j0 - 2*j1 - 2*j3]

V(J) matrix reshaped to show clearly symetry [A B; B A] in Matrix
[-j1 - j2  -j2 - j3     0         0           j1 + j3     ]
[                                                         ]
[-j2 - j3  -j2 - j3     0         0             0         ]
[                                                         ]
[   0         0      -j1 - j2  -j2 - j3       j1 + j3     ]
[                                                         ]
[   0         0      -j2 - j3  -j2 - j3         0         ]
[                                                         ]
[j1 - j3      0      j1 - j3      0      -j0 - 2*j1 - 2*j3]

'A' Matrix
[-j1 - j2  -j2 - j3]
[                  ]
[-j2 - j3  -j2 - j3]

'B' Matrix
[0  0]
[    ]
[0  0]

New V(J) Matrix (A - B)
[-j1 - j2  -j2 - j3]
[                  ]
[-j2 - j3  -j2 - j3]


---- Mineur Analysis ----
Limiting mineur analysis to mineurs of size smaller or equal to 5

Mineur:
Poly(j1*j2 + j1*j3 - j2*j3 - j3**2, j1, j2, j3, domain='ZZ')
Compounds number: [(0, 1)]

---- --------------- ----
Restrictions to solve
[j0 > 0, j1 > 0, j2 > 0, j3 > 0, -j1*j2 - j1*j3 + j2*j3 + j3**2 > 0]

Trying to find a solution with the restrictions

Found a solution on try number 1 of 1000
```

```
(Randomly) Chosen variable values:
[j3: 1.68144013191,
 j2: 0.876669794652,
 ]

Values that variables can take given the already chosen (see above) values for variables
[j3, j2]
[j1 = (0, 430130869234109/255810992655764)]

Sampling 10 points from js intervals
Saving sampled models to ‘samples/iwamoto_perfect‘
*************************
***  Iwamoto-Perfect  ***
*************************
```

The Iwamoto model under imperfect conditions

The input file

```
 # run with: "python -m listanalchem --model models/Iwamoto-Imperfect.py"
modelname = 'Iwamoto-Imperfect'
species = ["L", "D", "E-L", "E-D", "A"]
reactions = [
    " <-> A",           # 0 & 1.
    "A + L <-> 2L",     # 2 & 3.
    "A + L <-> L + D",  # 4 & 5.
    "A + D <-> 2D",     # 6 & 7.
    "A + D <-> D + L",  # 8 & 9.
    "L + E-L <-> ",     # 10 & 11.
    "D + E-D <-> ",     # 12 & 13.
    "D + E-L <-> ",     # 14 & 15.
    "L + E-D <-> ",     # 16 & 17.
    " -> E-L",          # 18.
    " -> E-D"           # 19.
]
dual_pairs = [(2,6),(4,8),(3,7),(5,9),(10,16),(12,14),(11,17),(13,15),(18,19)]

analyses = {
    "sna": {
        "enabled": False,
        "dual-pairs-in-ec": True,
        # "instability-heuristic": "trace-determinant",
        # "instability-heuristic": "characteristic-polynomial",
        "instability-heuristic": "mineurs",
        "sum-mineurs": True,
        "max-mineur-search-stop": 5,
        "simplification-tries": 10000,
        "num-samples": 10,
        "samples-folder": 'samples/iwamoto-imperfect',
    },
    "frank-pseudoquiral": {
```

```
        "enabled": True,
        "enantiomeric-pairs": [(0, 1), (2, 3)],
        "dual-pairs-in-ec": True,
        # "instability-heuristic": "trace-determinant",
        # "instability-heuristic": "characteristic-polynomial",
        "instability-heuristic": "mineurs",
        "sum-mineurs": True,
        "max-mineur-search-stop": 5,
        "simplification-tries": 10000,
        "num-samples": 10,
        "samples-folder": ’samples/iwamoto-imperfect’,
    }
}
```

The output file

```
 Welcome to Listanalchem!
Remember to check the model you’re loading, it must:
* Have a name ‘modelname‘
* Have a list of species ‘species‘ (note: the first two species will be considered
  enantiomers, all other species do not)
* Have a non-empty list of reactions

***************************
***  Iwamoto-Imperfect  ***
***************************

Species:
[’L’, ’D’, ’E-L’, ’E-D’, ’A’]
Reactions list:
[’ -> A’,
 ’A -> ’,
 ’L + A -> 2 L’,
 ’2 L -> L + A’,
 ’L + A -> L + D’,
 ’L + D -> L + A’,
 ’D + A -> 2 D’,
 ’2 D -> D + A’,
 ’D + A -> L + D’,
 ’L + D -> D + A’,
 ’L + E-L -> ’,
 ’ -> L + E-L’,
 ’D + E-D -> ’,
 ’ -> D + E-D’,
 ’D + E-L -> ’,
 ’ -> D + E-L’,
 ’L + E-D -> ’,
 ’ -> L + E-D’,
 ’ -> E-L’,
 ’ -> E-D’]
Dual pair equations (equations are numbered from 0 to 19):
```

```
[(2, 6),
 (4, 8),
 (3, 7),
 (5, 9),
 (10, 16),
 (12, 14),
 (11, 17),
 (13, 15),
 (18, 19)]
Regular reactions (not dual)
[0, 1]


Stoichiometric Matrix:
[[ 0  0  1 -1  0  0  0  0  1 -1 -1  1  0  0  0  0 -1  1  0  0]
 [ 0  0  0  0  1 -1  1 -1  0  0  0  0 -1  1 -1  1  0  0  0  0]
 [ 0  0  0  0  0  0  0  0  0  0 -1  1  0  0 -1  1  0  0  1  0]
 [ 0  0  0  0  0  0  0  0  0  0  0  0 -1  1  0  0 -1  1  0  1]
 [ 1 -1 -1  1 -1  1 -1  1 -1  1  0  0  0  0  0  0  0  0  0  0]]

Reactions Order Matrix:
[[0 0 1 2 1 1 0 0 0 1 1 0 0 0 0 0 1 0 0 0]
 [0 0 0 0 0 1 1 2 1 1 0 0 1 0 1 0 0 0 0 0]
 [0 0 0 0 0 0 0 0 0 0 0 1 0 0 0 1 0 0 0 0 0]
 [0 0 0 0 0 0 0 0 0 0 0 0 0 1 0 0 0 1 0 0 0]
 [0 1 1 0 1 0 1 0 1 0 0 0 0 0 0 0 0 0 0 0 0]]

Velocity Function:
[   k0    ]
[         ]
[  k1*x4  ]
[         ]
[k2*x0*x4 ]
[         ]
[      2  ]
[ k3*x0   ]
[         ]
[k4*x0*x4 ]
[         ]
[k5*x0*x1 ]
[         ]
[k6*x1*x4 ]
[         ]
[      2  ]
[ k7*x1   ]
[         ]
[k8*x1*x4 ]
[         ]
[k9*x0*x1 ]
[         ]
```

```
[k10*x0*x2]
[         ]
[   k11   ]
[         ]
[k12*x1*x3]
[         ]
[   k13   ]
[         ]
[k14*x1*x2]
[         ]
[   k15   ]
[         ]
[k16*x0*x3]
[         ]
[   k17   ]
[         ]
[   k18   ]
[         ]
[   k19   ]

Differential equations functions (polynomials) vector:
[                                                              2
[         -k10*x0*x2 + k11 - k16*x0*x3 + k17 + k2*x0*x4 - k3*x0  + k8*x1*x4 -
[
[
[         -k12*x1*x3 + k13 - k14*x1*x2 + k15 + k4*x0*x4 - k5*x0*x1 + k6*x1*x4
[
[                           -k10*x0*x2 + k11 - k14*x1*x2 + k15 + k18
[
[                           -k12*x1*x3 + k13 - k16*x0*x3 + k17 + k19
[
[                             2                                        2
[k0 - k1*x4 - k2*x0*x4 + k3*x0  - k4*x0*x4 + k5*x0*x1 - k6*x1*x4 + k7*x1  - k8

               ]
k9*x0*x1       ]
               ]
       2       ]
- k7*x1        ]
               ]
               ]
               ]
               ]
               ]
               ]
*x1*x4 + k9*x0*x1]


============================= ( - 6 - ) =============================
```

=== Algorithm based on the Frank inequality, according to reference [5] in README.md file ===

*** Solving semialgebraic problem (sampling from a non-linear inequalities indirectly) using Clarke factorization according to the Stoichiometric Network Analysis (SNA) ***

Enantiomers divided into L's and D's groups
(('L', 'E-L'), ('D', 'E-D'))

Adding rows to the Stoichiometric Matrix encoding the dual pairs conditions in order to make the cone defined by the Extreme Currents smaller and 'more precise'

Extended Stoichiometric matrix with rows indicating dual pairs
```
[[ 0.  0.  1. -1.  0.  0.  0.  0.  1. -1. -1.  1.  0.  0.  0.  0. -1.  1.
   0.  0.]
 [ 0.  0.  0.  0.  1. -1.  1. -1.  0.  0.  0.  0. -1.  1. -1.  1.  0.  0.
   0.  0.]
 [ 0.  0.  0.  0.  0.  0.  0.  0.  0.  0. -1.  1.  0.  0. -1.  1.  0.  0.
   1.  0.]
 [ 0.  0.  0.  0.  0.  0.  0.  0.  0.  0.  0. -1.  1.  0.  0. -1.  1.
   0.  1.]
 [ 1. -1. -1.  1. -1.  1. -1.  1. -1.  1.  0.  0.  0.  0.  0.  0.  0.  0.
   0.  0.]
 [ 0.  0.  1.  0.  0.  0. -1.  0.  0.  0.  0.  0.  0.  0.  0.  0.  0.  0.
   0.  0.]
 [ 0.  0.  0.  0.  1.  0.  0.  0. -1.  0.  0.  0.  0.  0.  0.  0.  0.  0.
   0.  0.]
 [ 0.  0.  0.  1.  0.  0.  0. -1.  0.  0.  0.  0.  0.  0.  0.  0.  0.  0.
   0.  0.]
 [ 0.  0.  0.  0.  0.  1.  0.  0.  0. -1.  0.  0.  0.  0.  0.  0.  0.  0.
   0.  0.]
 [ 0.  0.  0.  0.  0.  0.  0.  0.  0.  0.  1.  0.  0.  0.  0.  0. -1.  0.
   0.  0.]
 [ 0.  0.  0.  0.  0.  0.  0.  0.  0.  0.  0.  0.  1.  0. -1.  0.  0.  0.
   0.  0.]
 [ 0.  0.  0.  0.  0.  0.  0.  0.  0.  0.  0.  1.  0.  0.  0.  0.  0. -1.
   0.  0.]
 [ 0.  0.  0.  0.  0.  0.  0.  0.  0.  0.  0.  0.  0.  1.  0. -1.  0.  0.
   0.  0.]
 [ 0.  0.  0.  0.  0.  0.  0.  0.  0.  0.  0.  0.  0.  0.  0.  0.  0.  0.
   1. -1.]]
```

Extreme Currents Matrix (computed from Stoichiometric Matrix)
```
[[1 0 0 0 0 0 0 4 4]
 [1 0 0 0 0 0 0 0 0]
 [0 1 0 1 0 0 0 2 0]
 [0 1 1 0 0 0 0 0 0]
 [0 0 1 0 1 0 0 0 2]
 [0 0 0 1 1 0 0 0 0]
 [0 1 0 1 0 0 0 2 0]
 [0 1 1 0 0 0 0 0 0]
```

```
 [0 0 1 0 1 0 0 0 2]
 [0 0 0 1 1 0 0 0 0]
 [0 0 0 0 0 0 1 1 1]
 [0 0 0 0 0 0 1 0 0]
 [0 0 0 0 0 1 0 1 1]
 [0 0 0 0 0 1 0 0 0]
 [0 0 0 0 0 1 0 1 1]
 [0 0 0 0 0 1 0 0 0]
 [0 0 0 0 0 0 1 1 1]
 [0 0 0 0 0 0 1 0 0]
 [0 0 0 0 0 0 0 2 2]
 [0 0 0 0 0 0 0 2 2]]

E_omega column
[j0 + 4*j7 + 4*j8]
[                ]
[       j0       ]
[                ]
[ j1 + j3 + 2*j7 ]
[                ]
[    j1 + j2     ]
[                ]
[ j2 + j4 + 2*j8 ]
[                ]
[    j3 + j4     ]
[                ]
[ j1 + j3 + 2*j7 ]
[                ]
[    j1 + j2     ]
[                ]
[ j2 + j4 + 2*j8 ]
[                ]
[    j3 + j4     ]
[                ]
[  j6 + j7 + j8  ]
[                ]
[       j6       ]
[                ]
[  j5 + j7 + j8  ]
[                ]
[       j5       ]
[                ]
[  j5 + j7 + j8  ]
[                ]
[       j5       ]
[                ]
[  j6 + j7 + j8  ]
[                ]
[       j6       ]
[                ]
```

```
[  2*j7 + 2*j8   ]
[                ]
[  2*j7 + 2*j8   ]

V(J) Matrix
[ -j1 - 2*j2 - j4 - 2*j6 - 2*j8           j2 - j3 + 2*j8               -j6 - j
[
[        j2 - j3 + 2*j8            -j1 - 2*j2 - j4 - 2*j5 - 2*j8       -j5 - j
[
[         -j6 - j7 - j8                    -j5 - j7 - j8           -j5 - j6 -
[
[         -j6 - j7 - j8                    -j5 - j7 - j8                     0
[
[j1 + j2 + j3 + j4 - 2*j7 - 2*j8  j1 + j2 + j3 + j4 - 2*j7 - 2*j8            0

7 - j8           -j6 - j7 - j8             j1 + j2 + j3 + j4 + 2*j7 + 2*j8

7 - j8           -j5 - j7 - j8             j1 + j2 + j3 + j4 + 2*j7 + 2*j8

2*j7 - 2*j8            0                                  0

             -j5 - j6 - 2*j7 - 2*j8                       0

                       0            -j0 - 2*j1 - 2*j2 - 2*j3 - 2*j4 - 4*j7 -

    ]
    ]
    ]
    ]
    ]
    ]
    ]
    ]
4*j8]

V(J) matrix reshaped to show clearly symetry [A B; B A] in Matrix
[ -j1 - 2*j2 - j4 - 2*j6 - 2*j8       -j6 - j7 - j8              j2 - j3 + 2*
[
[         -j6 - j7 - j8           -j5 - j6 - 2*j7 - 2*j8          -j5 - j7 -
[
[        j2 - j3 + 2*j8                -j5 - j7 - j8       -j1 - 2*j2 - j4 - 2
[
[         -j6 - j7 - j8                      0                    -j5 - j7 -
[
[j1 + j2 + j3 + j4 - 2*j7 - 2*j8            0             j1 + j2 + j3 + j4 -

j8               -j6 - j7 - j8             j1 + j2 + j3 + j4 + 2*j7 + 2*j8

j8                     0                                  0

```

```
*j5 - 2*j8       -j5 - j7 - j8              j1 + j2 + j3 + j4 + 2*j7 + 2*j8

j8           -j5 - j6 - 2*j7 - 2*j8                       0

2*j7 - 2*j8            0             -j0 - 2*j1 - 2*j2 - 2*j3 - 2*j4 - 4*j7 -

    ]
    ]
    ]
    ]
    ]
    ]
    ]
    ]
4*j8]

'A' Matrix
[-j1 - 2*j2 - j4 - 2*j6 - 2*j8      -j6 - j7 - j8     ]
[                                                     ]
[        -j6 - j7 - j8          -j5 - j6 - 2*j7 - 2*j8]

'B' Matrix
[j2 - j3 + 2*j8  -j6 - j7 - j8]
[                             ]
[-j5 - j7 - j8         0      ]

New V(J) Matrix (A - B)
[-j1 - 3*j2 + j3 - j4 - 2*j6 - 4*j8           0          ]
[                                                        ]
[             j5 - j6               -j5 - j6 - 2*j7 - 2*j8]


---- Mineur Analysis ----
Limiting mineur analysis to mineurs of size smaller or equal to 5

Mineur:
Poly(j1 + 3*j2 - j3 + j4 + j5 + 3*j6 + 2*j7 + 6*j8, j1, j2, j3, j4, j5, j6, j7
, j8, domain='ZZ')
Compounds number: [(0,), (1,)]

Mineur:
Poly(j1*j5 + j1*j6 + 2*j1*j7 + 2*j1*j8 + 3*j2*j5 + 3*j2*j6 + 6*j2*j7 + 6*j2*j8
 - j3*j5 - j3*j6 - 2*j3*j7 - 2*j3*j8 + j4*j5 + j4*j6 + 2*j4*j7 + 2*j4*j8 + 2*j
5*j6 + 4*j5*j8 + 2*j6**2 + 4*j6*j7 + 8*j6*j8 + 8*j7*j8 + 8*j8**2, j1, j2, j3,
j4, j5, j6, j7, j8, domain='ZZ')
Compounds number: [(0, 1)]

---- -------------- ----
Restrictions to solve
[j0 > 0,
```

```
 j1 > 0,
 j2 > 0,
 j3 > 0,
 j4 > 0,
 j5 > 0,
 j6 > 0,
 j7 > 0,
 j8 > 0,
 -j1 - 3*j2 + j3 - j4 - j5 - 3*j6 - 2*j7 - 6*j8 > 0,
 -j1*j5 - j1*j6 - 2*j1*j7 - 2*j1*j8 - 3*j2*j5 - 3*j2*j6 - 6*j2*j7 - 6*j2*j8 + j3*j5 +
  j3*j6 + 2*j3*j7 + 2*j3*j8 - j4*j5 - j4*j6 - 2*j4*j7 - 2*j4*j8 - 2*j5*j6 - 4*j5*j8 -
  2*j6**2 - 4*j6*j7 - 8*j6*j8 - 8*j7*j8 - 8*j8**2 > 0]

Trying to find a solution with the restrictions

Found a solution on try number 4 of 10000

(Randomly) Chosen variable values:
[j6: 1.62297451374,
 j8: 0.945971762709,
 j5: 0.910672504215,
 j7: 1.9686725392,
 ]

Values that variables can take given the already chosen (see above) values for variables
[j8, j6, j5, j7]
[j1 = (0, oo)
 j2 = (0, oo)
 j3 = (Max(j1 + 3*j2 + 153927717000673/10000000000000, j1 +
 2508880686527950*j2/836293562175983 + 5879006655441000/836293562175983), oo)
 j4 = (0, Min(-j1 - 2508880686527950*j2/836293562175983 + j3 - 5879006655441000/836293562175983,
 -j1 - 3*j2 + j3 - 153927717000673/10000000000000))]

Sampling 10 points from js intervals
Saving sampled states to ‘samples/iwamoto-imperfect‘
**************************
***  Iwamoto-Imperfect  ***
**************************
```

The Iwamoto model under imperfect conditions figure

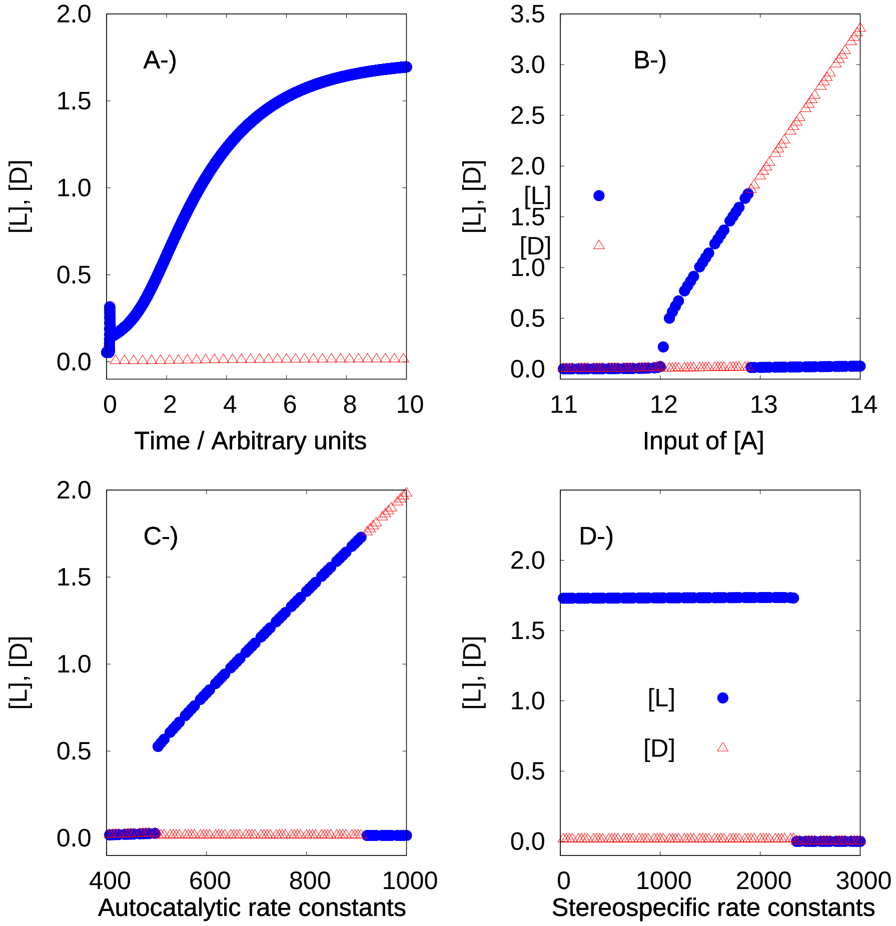

Figure S1. The Iwamoto model under imperfect conditions. A-) Time series; and bifurcation diagrams for B-) Input flow reaction $P \xrightarrow{k_0} A$, C-) autocatalytic reactions $A + L \xrightarrow{k_2} 2\,L$ and $A + D \xrightarrow{k_2} 2\,D$, and D-) stereospecific reactions $L + E_L \xrightarrow{k_6} Z_L$ and $D + E_D \xrightarrow{k_8} Z_D$. The initial concentrations of the enantiomers L and D were equals until the 15th decimal position, equivalent to an enantiomeric excess $ee = \dfrac{[L] - [D]}{[L] + [D]} = 7.051936 \times 10^{-15}$.
